# Protective effects of herbal toothpastes against dentin erosion: an in vitro study

**DOI:** 10.1038/s41598-026-54828-y

**Published:** 2026-05-25

**Authors:** Alara Aksoy, Nazmiye Donmez, Ceren Deger

**Affiliations:** 1 Department of Restorative Dentistry, Institute of Health Sciences, Bezmialem Vakıf University, Fatih, Istanbul, 34093 Türkiye; 2 Department of Restorative Dentistry, Faculty of Dentistry, Bolu Abant Izzet Baysal University, Bolu, Türkiye; 3 Department of Restorative Dentistry, Faculty of Dentistry, Bezmialem Vakıf University, Istanbul, Türkiye

**Keywords:** Atomic force microscopy, Dentin, Remineralization, Scanning electron microscopy, Tooth erosion, Toothpastes, Health care, Materials science, Medical research

## Abstract

This in vitro study evaluated the protective effects of different herbal and bioactive toothpaste formulations on dentin surfaces exposed to erosive challenges. A total of 96 dentin specimens were prepared from 48 human molars and randomly assigned to six groups (*n* = 16), each receiving one of the following treatments: fluoride-containing toothpaste, clove-containing toothpaste, propolis-containing toothpaste, fluoride combined with nano-hydroxyapatite (F+nHAp), nano-hydroxyapatite toothpaste (nHAp), and casein phosphopeptide–amorphous calcium phosphate (CPP-ACP) based toothpaste. The specimens underwent repeated erosion–brushing–remineralization cycles using 0.01 M hydrochloric acid (pH 1.5) to simulate intrinsic erosive conditions. Surface roughness and microhardness were measured at baseline, after demineralization, and following remineralization. Additional surface characterization was conducted using scanning electron microscopy (SEM) and atomic force microscopy (AFM). All groups showed increased surface roughness after demineralization. However, only the clove group exhibited a significant reduction in roughness after remineralization (*p* < 0.05), returning to baseline levels. Microhardness significantly decreased in all groups following demineralization (*p* < 0.05). After remineralization, only the clove, F+nHAp, and nHAp groups regained microhardness values comparable to baseline (*p* > 0.05). These findings demonstrate that clove-containing, nano-hydroxyapatite-containing, and fluoride+nano-hydroxyapatite toothpaste formulations were the most effective in preserving dentin surface integrity, suggesting their potential role in the prevention of dentin erosion.

## Introduction

Dental erosion is defined as the progressive and irreversible loss of dental hard tissues induced by chemical processes involving acids, in the absence of bacterial activity^1^. The condition is primarily driven by extrinsic and intrinsic acid sources that lower the pH of the oral environment, initiating demineralization^2,3^.

Extrinsic acids contributing to dental erosion are typically derived from dietary sources such as acidic beverages, foods, and certain medications, whereas intrinsic acids originate from gastric sources, most notably gastroesophageal reflux and recurrent vomiting episodes^4^.

Among these, gastroesophageal reflux disease (GERD) represents a significant intrinsic etiological factor, as gastric contents may reach the oral cavity during reflux events, exposing dental tissues to hydrochloric acid with a pH of approximately 1–2. Consequently, clinical studies have reported a higher prevalence of dental erosion in individuals affected by GERD compared with healthy populations^3^.

Repeated exposure of teeth to gastric acids can lead to progressive demineralization of dental hard tissues, thereby increasing their susceptibility to mechanical wear and erosion^2,3^.

Dental hard tissues are continuously exposed to cycles of demineralization and remineralization within the oral environment, and the presence of acidic challenges may disrupt this dynamic balance. When this dynamic balance shifts toward demineralization, progressive structural degradation of enamel and dentin occurs^5^. Due to its lower mineral content and tubular microstructure, dentin is particularly vulnerable to acid-induced damage compared with enamel^4^. As a result, erosion-related dentin loss may lead to hypersensitivity, pain, and functional impairment. Consequently, current management strategies emphasize preventive and non-invasive approaches rather than extensive restorative interventions^2,6^.

A growing body of evidence highlights the clinical relevance of non-invasive strategies in enhancing the remineralization of eroded dentin surfaces^7^. Various formulations have been developed to promote tissue repair, including bioactive compounds like casein phosphopeptide-amorphous calcium phosphate (CPP–ACP), nano-hydroxyapatite (nano-HA), and other calcium-based agents. Among these, CPP–ACP enhances remineralization by creating a supersaturated calcium-phosphate reservoir within the oral cavity, thereby inhibiting demineralization^8^. Tooth Mousse (MI Paste), a commercial product containing 10% CPP–ACP, has demonstrated promising outcomes in maintaining intraoral pH balance and promoting enamel and dentin remineralization^9^.

Similarly, nano-hydroxyapatite (Ca₁₀[PO₄]₆[OH]₂), the principal mineral component of enamel and dentin, has gained substantial attention due to its biomimetic properties, biocompatibility, and ability to integrate with tooth structure^10^. Products such as ApaCare^®^ toothpaste, which contain medical-grade hydroxyapatite, are marketed for their potential to restore enamel integrity, alleviate dentin hypersensitivity, and provide a protective barrier following acidic challenges^11^.

In parallel with these synthetic formulations, herbal-based toothpastes have garnered interest as potential alternatives, particularly among populations seeking natural products free from synthetic additives^12^. These formulations often incorporate bioactive plant-derived compounds with antimicrobial, anti-inflammatory, and antioxidant properties^13^. Notably, clove (Syzygium aromaticum) and propolis have emerged as prominent ingredients in this category.

Clove, rich in eugenol, possesses well-documented analgesic, antiseptic, and anti-inflammatory effects. Its inclusion in oral care products is supported by evidence demonstrating reductions in dentin hypersensitivity and inhibition of oral pathogens^14^.

Previous studies reported that clove and green tea extracts demonstrated protective effects against dentin erosion, supporting the potential of herbal ingredients in erosion prevention^14^. Another well-studied natural product is propolis, a resinous substance produced by honeybees. It contains more than 300 bioactive compounds, including flavonoids, phenolic acids, and esters, with notable antioxidant, antimicrobial, and anti-inflammatory effects, primarily attributed to its high flavonoid content^13,15^.

Despite promising in vitro and in vivo data on enamel remineralization^16,17^, the effects of herbal formulations on dentin erosion remain inadequately characterized. A limited number of studies have explored their potential to mitigate dentin demineralization and promote surface recovery^14,16–18^.

Therefore, the present in vitro study aimed to evaluate the protective effects of various toothpaste formulations including herbal, nano-hydroxyapatite, and CPP–ACP-based products on erosion-exposed human dentin. The study specifically assessed changes in dentin surface microhardness and surface roughness following erosive challenges. The null hypothesis was that herbal toothpaste formulations would not produce statistically significant differences in dentin surface microhardness or surface roughness of eroded dentin.

## Methods

A literature review was conducted to determine the minimum required sample size before the initiation of the study. Based on a Type I error probability (α) of 0.05, a statistical power (1–β) of 0.85, and an effect size of 0.25, it was calculated that a minimum sample size of 30 would be sufficient. However, to increase the statistical reliability of the study, specimens obtained from 48 extracted human molars were included (*n* = 16 per group). Power analysis was performed using G*Power version 3.1. All methods were performed in accordance with the relevant guidelines and regulations. This in vitro study received ethical approval from the Bezmialem Vakıf University Non-Interventional Clinical Research Ethics Committee (Approval No: 2022/409). Verbal informed consent was obtained from all donors prior to tooth extraction.

In this study, a total of 48 human molar teeth were used, extracted for periodontal reasons. Teeth exhibiting cracks, fractures, carious lesions, or other structural defects were excluded. Following extraction, periodontal debris was carefully removed using an ultrasonic scaler (Woodpecker UDS-E LED, Guilin, China), and the teeth were subsequently polished with a prophylactic paste (Promida Dental, Eskisehir, Türkiye). Specimens without visible defects were stored in 0.1% thymol solution for up to two months before experimentation. The thymol solution was renewed weekly to maintain antimicrobial effectiveness.

Following removal from the thymol solution, the teeth were rinsed with distilled water. Under water-cooled conditions, each tooth was sectioned mesiodistally using a low-speed microtome (Mecatome T180, PRESI, France) to obtain the buccal and lingual surfaces. The roots were then separated at the enamel–cementum junction. A total of 96 dentin specimens were prepared. The crowns were embedded in self-curing acrylic resin (BLAU CRYL, Bursa, Türkiye) using Teflon molds, with the vestibular surfaces oriented outward in accordance with the manufacturer’s instructions. The buccal surfaces were abraded with 180-grit silicon carbide abrasive paper using an automatic polishing device (Minitech 233, PRESI, Eybens, France) to expose the dentin layer. Subsequently, the exposed dentin surfaces were sequentially polished with 400-, 600-, 800-, 1000-, 1200-, and 1500-grit silicon carbide papers under continuous water irrigation.

After numbering, the surface roughness of each specimen was measured at baseline (T0) at three distinct locations using a contact profilometer (Mahr M300C, Carl-Mahr, Germany), and the arithmetic mean of these values was calculated. Subsequently, microhardness was determined at baseline (T0) at three different points using a Vickers hardness tester (Shimadzu HMV, Kyoto, Japan) under a load of 0.2 N applied for 10 s. The mean value was recorded as the representative microhardness for each specimen. To prevent dehydration and minimize potential variation in results, all specimens were stored at room temperature in sealed containers filled with distilled water throughout the experimental period.

Six distinct toothpaste formulations were applied to the specimen surfaces, with each group comprising 16 specimens (*n* = 16):

Fluoride Group (Positive Control): Fluoride-containing toothpaste (Colgate, Palmolive, New York, USA).

Clove Group: Clove-containing toothpaste (Eyüp Sabri Tuncer, Istanbul, Türkiye).

Propolis Group: Propolis-containing toothpaste (Eyüp Sabri Tuncer, Istanbul, Türkiye).

F+nHAp Group: Fluoride and nano-hydroxyapatite-containing toothpaste (ApaCare, Cumdente GmbH, Tübingen, Germany).

nHAp Group: Nano-hydroxyapatite-containing toothpaste (PrevDent, Riga, Latvia).

CPP-ACP Group: Casein phosphopeptide–amorphous calcium phosphate-containing toothpaste (Tooth Mousse, GC Corporation, Tokyo, Japan).

The complete ingredient lists of all tested toothpastes were obtained from the manufacturers’ product information and are provided in Table 1.

**Table 1:** Composition of the toothpastes used in the study.

No	Toothpaste	Manufacturer	Chemical Composition
1	Fluoride-containing toothpaste (F) (Positive Control)	Colgate, New York, USA	Contains Sodium Monofluorophosphate (1450 ppm F), Calcium Carbonate, Aqua, Sorbitol, Sodium Lauryl Sulfate, Hydrated Silica, Arginine, Aroma, Cellulose Gum, Sodium Carbonate, Benzyl Alcohol, Phosphoric Acid, Sodium Saccharin, Sodium Bicarbonate, Cl 77,891
2	Clove-containing toothpaste (C)	Eyüp Sabri Tuncer, Istanbul, Türkiye	Aqua, Sorbitol, Hydrated Silica, Glycerin, Xylitol, Polysorbate 20, Cocamidopropyl Betaine, Disodium Phosphate, Eugenia Caryophyllus Flower Oil, Xanthan Gum, Rebaudioside A, Phenylpropanol, Propanediol, Menthol, Caprylyl Glycol, Thymus Vulgaris Leaf Oil, Melaleuca Alternifolia Leaf Oil, Tocopherol
3	Propolis-containing toothpaste (P)	Eyüp Sabri Tuncer, Istanbul, Türkiye	Sorbitol, Aqua, Calcium Carbonate, Hydrated Silica, Glycerin, Xylitol, Polysorbate 20, Cocamidopropyl Betaine, Disodium Phosphate, Xanthan Gum, Aroma, Phenylpropanol, Caprylyl Glycol, Stevia Rebaudiana Extract, Propolis Extract, Mentha Piperta Oil, Menthol, Thymus Vulgaris Leaf Oil
4	Fluoride + nano-hydroxyapatite-containing toothpaste (F+nHAp)	Apa Care, Tübingen, Germany	Aqua, Hydrated Silica, Sorbitol, Propylene Glycol, Glycerin, Sodium C14-16 Olefin Sulfonate, Hydroxyapatite, Aroma, Cellulose Gum, CI 77,891, Sodium Fluoride, Allantoin, Sodium Saccharin, Tetrapotassium Pyrophosphate, Limonene
5	Nano-hydroxyapatite-containing toothpaste (nHAp)	PrevDent, Riga, Latvia	Aqua, Hydrated Silica, Sorbitol, Glycerin, Xylitol, Potassium Nitrate, Nano-hydroxyapatite concentration 15%, Magnesium Aluminum Silicate, Mentha Piperita Oil, Sodium Lauroyl Sarcosinate, Xanthan Gum, Phenoxyethanol, Potassium Chloride, Sodium Sulfate, Sodium Saccharin, CI 77,891
6	Casein phosphopeptide amorphous calcium phosphate-containing toothpaste (CPP-ACP)	Tooth Mousse, Tokyo, Japan	Pure Water, Glycerol, RECALDENT (CPP-ACP), D-sorbitol, CMC-Na, Propylene glycol, Silicon dioxide, Titanium dioxide, Xylitol, Phosphoric acid, Flavoring, Zinc oxide, Guar gum, Propyl p-hydroxybenzoate, Butyl p-hydroxybenzoate

The Relative Dentin Abrasivity (RDA) value was available only for the ApaCare toothpaste according to the information provided by the manufacturer. For the other tested toothpastes, RDA values could not be identified from the manufacturers’ technical documentation or publicly available sources. Following the erosion cycle, one specimen from each group was selected for atomic force microscopy (AFM) analysis, and one specimen from each group was allocated for scanning electron microscopy (SEM) evaluation.

### Erosion cycle

The surface of each specimen was divided into two equal regions, designated as Area 1 and Area 2. The specimens were immersed in a 0.01 M hydrochloric acid solution (pH 1.5) for 30 s to induce erosion, then rinsed with distilled water and gently dried with a paper towel. Area 1 was subsequently covered with acid-resistant isolation tape. The protected area served as an internal reference region and was shielded from the brushing procedure, allowing evaluation of erosion alone while the exposed area was subjected to erosion and abrasion^19^. Area 2 was then brushed for one minute using a toothpaste-to-distilled water mixture at a 1:3 weight ratio. Following brushing, the specimens were rinsed again, dried, and incubated for 2 h at 37 °C in artificial saliva (pH 7.2)^20^, which consisted of 5 mM HEPES, 2.5 mM CaCl₂, 0.05 mM ZnCl₂, 0.68 mM KH₂PO₄, 30 mM KCl, and 120 mM NaCl^21^.

**Fig. 1:**
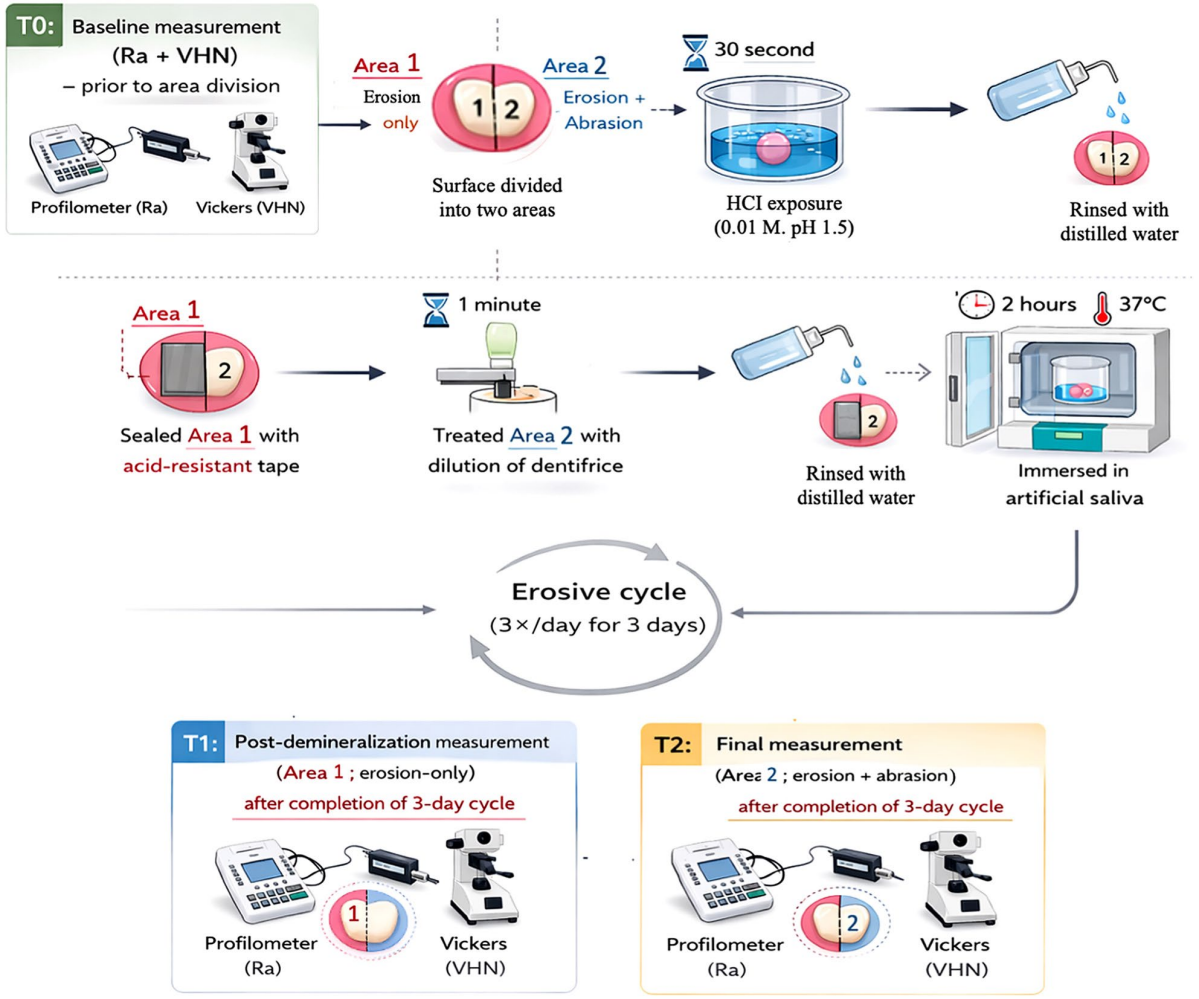
Schematic representation of specimen preparation, erosion cycles, and brushing procedures.

This entire erosion-remineralization cycle was repeated three times daily over a three-day period (Fig. 1)^20^.

At the end of the cycle, all specimens underwent ultrasonic cleaning for five minutes to eliminate any residual toothpaste.

Surface roughness and microhardness measurements were performed at three predefined time points: T0 (baseline, prior to the erosive challenge), T1 (post-demineralization, immediately after completion of the final acid exposure and before remineralization incubation), and T2 (post-remineralization, after completion of the final erosion–remineralization cycle).

T1 measurements were obtained from Area 1 after completion of the 3-day erosive protocol, immediately following the final acid exposure and prior to any brushing or remineralization procedure. Since Area 1 had been protected from brushing throughout the experiment, these values represent erosion-only effects without abrasion-related surface loss.

T2 measurements were subsequently recorded from Area 2 after completion of the full 3-day erosion–abrasion–remineralization cycle.

### Electric tooth brushing simulator

In this in vitro study, an electric toothbrush (Braun Oral-B Advance Power) equipped with an Oral-B Cross Action brush head (Procter & Gamble Sales Distribution Ltd., Istanbul, Türkiye) was used in continuous mode. The device was mounted on a custom-made holder delivering a constant brushing force of 2 N. Brushing cycles were performed three times daily for three consecutive days, resulting in a total brushing duration of nine minutes per specimen, simulating approximately four months of clinical brushing^22^. After each brushing session, the dentifrice mixture was refreshed, and all specimens were thoroughly rinsed to eliminate residual material.

### Surface characterization

#### Surface roughness measurement

In all groups, surface roughness (Ra) was measured using a contact profilometer (Mahr M300C, Carl-Mahr, Göttingen, Germany) at three separate points for each specimen, individually for both the Area 1 and Area 2. A 5 μm tip-radius stylus was used, and the arithmetic mean of the three measurements was calculated and recorded for each specimen.

#### Microhardness measurement

Vickers microhardness was measured at three separate points for both the Area 1 and Area 2 using a microhardness tester (Shimadzu HMV, Japan). The average of the three measurements was recorded as the representative microhardness value for each specimen.

### Statistical analysis

Analyses were performed using technical replicates, with three measurements taken for both surface roughness and microhardness from each specimen. Descriptive statistics (mean ± standard deviation) were calculated for all variables. The Shapiro-Wilk test was used to assess the normality of the data before hypothesis testing. A two-factor within-subject ANOVA was performed to assess the effects of toothpaste type and experimental phase (baseline, post-demineralization, and post-remineralization) on surface roughness and microhardness.

A paired t-test with Bonferroni correction was used to identify significant differences in the group(s). Statistical significance was set at *p* < 0.05. Exact p-values, test statistics, degrees of freedom, and partial eta-squared (η²) effect sizes are reported where appropriate. Statistical analyses were performed using R (version 4.3.0) software.

### AFM analysis

Atomic force microscopy (AFM) analysis was performed on specimens from each group using a silicon probe-equipped device (Quesant Instrument Corp., Agoura Hills, CA). Measurements included root mean square roughness (R < sub> rms</sub> ) and maximum depth (R < sub> valley</sub> ), reported in micrometers (µm). All scans were conducted in non-contact mode using a piezoelectric tip over a standardized 10 μm × 10 μm area along the z-axis. AFM images were captured at a resolution of 256 × 256 pixels.

### SEM analysis

To ensure adequate electrical conductivity for SEM analysis, one specimen from each group was coated with a 10 nm gold–palladium layer using a sputter coater (SC7640 Mini, Quorum Technologies, UK) under anoxic conditions. The coated specimens were then examined using the scanning electron microscope (Zeiss EVO LS 10, Carl Zeiss, Jena, Germany) at ×5000 magnification. SEM images were obtained at three predefined stages: baseline (prior to the erosive challenge), after completion of the full 3-day experimental protocol from Area 1 (erosion-only region), and after completion of the same protocol from Area 2 (erosion + abrasion + remineralization region). No SEM imaging was performed at intermediate stages of the experimental cycle.

## Results

### Surface roughness analysis findings

A two-way repeated-measures ANOVA revealed significant main effects of toothpaste type (F(5, 75) = 5.289, *p* = 0.000324) and experimental phase (F(2, 30) = 352.5, *p* < 0.0001) on surface roughness, as well as a significant interaction between these factors (F(10, 150) = 3.111, *p* = 0.00124). The surface roughness values obtained from each group are presented in Table 2.

**Table 2:** Mean surface roughness (Ra, µm) values of all toothpaste groups across the experimental phases.

Phase	Fluoride Group	Clove Group	Propolis Group	F+nHAp Group	nHAp Group	CPP-ACP Group	*P*-value
Baseline	0.120 ± 0.036^ab^	0.129 ± 0.083^ab^	0.098 ± 0.037^b^	0.099 ± 0.03^b^	0.111 ± 0.053^ab^	0.135 ± 0.051^ab^	0.00124
Demineralization	0.333 ± 0.133^cd^	0.325 ± 0.135^cd^	0.403 ± 0.072^cd^	0.355 ± 0.08^c^	0.420 ± 0.068^cd^	0.312 ± 0.076^c^	
Remineralization	0.377 ± 0.151^cd^	0.288 ± 0.144^ac^	0.463 ± 0.046^d^	0.352 ± 0.118^cd^	0.443 ± 0.099^cd^	0.364 ± 0.104^cd^	

There is a statistically significant difference between the values of group-phase combinations that do not have a common superscript (*p* < 0.05). P-value was obtained by a two-factor repeated measures ANOVA test. To compare the means of group-phase combinations (post-hoc test), a paired t-test with Bonferroni correction was used.

**Fig. 2:**
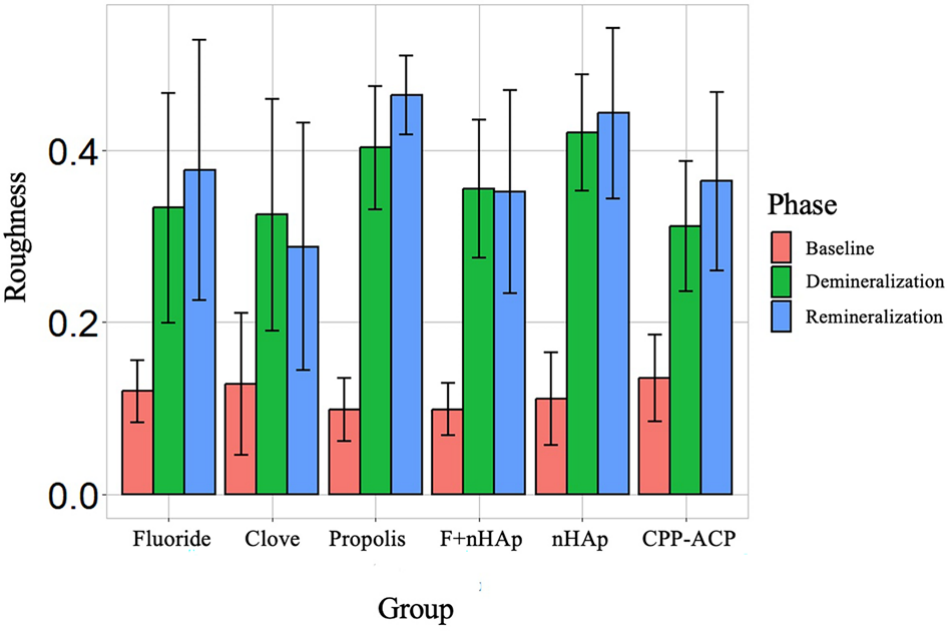
Surface roughness values (Ra, µm) of all toothpaste groups after each experimental phase. Values are presented as mean ± standard deviation (SD).

Figure 2 illustrates the changes in surface roughness across the experimental phases for the different toothpaste groups.

In the fluoride, propolis, F+nHAp, nHAp, and CPP–ACP groups, surface roughness values increased significantly after both demineralization and remineralization compared to baseline values (*p* = 0.0008). However, no statistically significant differences were observed between the demineralization and remineralization phases within these groups (*p* > 0.05).

In the clove group, surface roughness increased significantly after demineralization (*p* = 0.002), but decreased numerically following remineralization, returning to values statistically comparable to baseline. No statistically significant difference was observed between the demineralization and remineralization phases (*p* > 0.05).

In the CPP–ACP group, surface roughness values in the demineralization and remineralization phases were significantly higher compared to the baseline (*p* = 0.013). However, no statistically significant difference was observed between the demineralization and remineralization phases (*p* > 0.05).

When comparing changes across groups, only the clove group demonstrated a statistically significant decrease in roughness after remineralization compared to the demineralized state (*p* = 0.003). In contrast, all other groups exhibited roughness values post-remineralization that remained significantly higher than baseline (*p* = 0.027).

In the intergroup comparison of surface roughness values after the remineralization phase, a statistically significant difference was observed between the clove and propolis groups (*t*(15)= − 6.94, *p* = 4.74 × 10⁻⁶), with the propolis group exhibited the highest and the clove group the lowest values.

A two-way repeated-measures ANOVA for surface microhardness showed significant main effects of toothpaste type (F(5, 75) = 34.43, *p* < 0.0001) and experimental phase (F(2, 30) = 1018, *p* < 0.0001), as well as a significant interaction between these factors (F(10, 150) = 24.79, *p* < 0.0001).

### Surface microhardness analysis findings

**Fig. 3:**
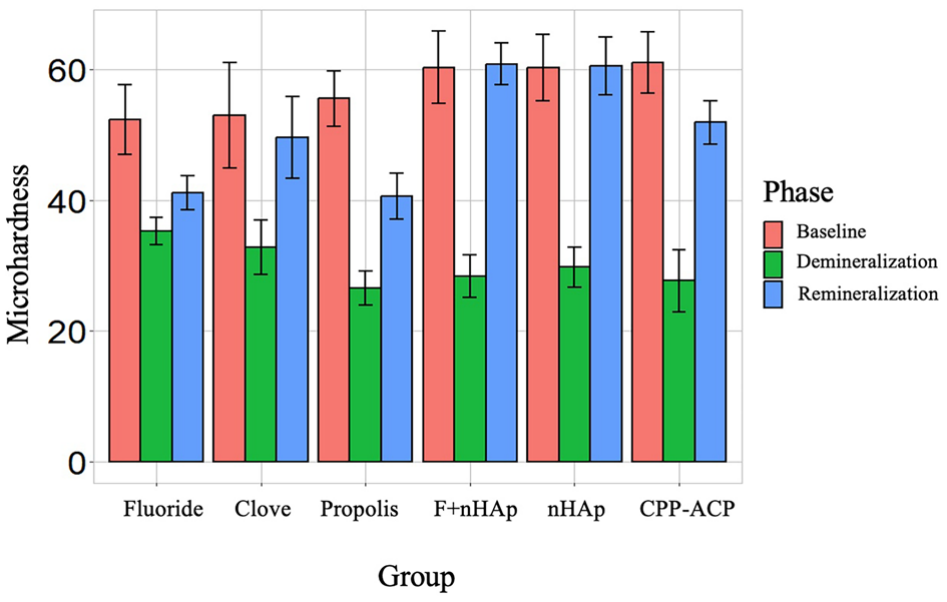
Microhardness values (VHN) of all toothpaste groups after each experimental phase. Values are presented as mean ± standard deviation (SD).

Changes in microhardness across the experimental phases for the different toothpaste groups are illustrated in Fig. 3. Table 3 presents the mean (± standard deviation) surface microhardness values for all toothpaste groups after demineralization and remineralization, along with intra-group comparisons. A statistically significant reduction in microhardness was observed in all groups following demineralization compared to the baseline values (*p* = 0.0002).

**Table 3:** Mean surface microhardness (VHN) values of the toothpaste groups across the experimental phases.

Phase	Fluoride Group	Clove Group	Propolis Group	F+nHAp Group	nHAp Group	CPP-ACP Group	*P*-value
Baseline	52.36 ± 5.33^ab^	53.02 ± 8.06^abf^	55.56 ± 4.24^abf^	60.30 ± 5.54^f^	60.3 ± 5.05^f^	61.02 ± 4.69^bf^	< 0.001
Demineralization	35.31 ± 2.07^c^	32.81 ± 4.20^cg^	26.50 ± 2.62^h^	28.35 ± 3.28^gh^	29.76 ± 3.03^gh^	27.66 ± 4.77^gh^	
Remineralization	41.15 ± 2.56^de^	49.63 ± 6.27^ad^	40.61 ± 3.53^e^	60.87 ± 3.18^f^	60.52 ± 4.44^bf^	51.92 ± 3.34^a^	

P-values were obtained using two-way repeated-measures ANOVA followed by Bonferroni-corrected paired t-tests. Significant main effects and interactions were detected for toothpaste type and experimental phase. Values that do not share a common letter within the same row or column differ significantly (*p* < 0.05).

Following remineralization, all groups exhibited a statistically significant increase in microhardness values compared to their respective post-demineralization measurements (*p* = 0.0005). However, when compared to baseline values, a significant reduction in final microhardness was observed in the fluoride (*p* = 0.015), propolis (*p* = 0.008), and CPP–ACP (*p* < 0.001) groups. In contrast, no significant differences were found between baseline and post-remineralization values in the clove, F+nHAp, and nHAp groups (*p* > 0.05), indicating effective recovery.

In the fluoride group, microhardness values decreased significantly after both demineralization and remineralization compared to baseline (*p* = 0.012), although remineralization resulted in a significant improvement over the demineralized state (*p* = 0.021).

In the clove group, demineralization led to a significant reduction in microhardness (*p* < 0.05), while remineralization restored it to baseline levels (*p* > 0.05), showing a significant improvement from the demineralized state (*p* = 0.019).

The propolis group showed a significant drop in microhardness after demineralization and a partial recovery after remineralization (both *p* = 0.030); however, post-remineralization values remained below baseline (*p* < 0.05).

In the F+nHAp and nHAp groups, demineralization significantly reduced microhardness (*p* < 0.05), but remineralization restored it to levels statistically similar to baseline (*p* > 0.05).

The CPP–ACP group experienced the greatest loss of microhardness, with significantly lower values than baseline after both demineralization and remineralization (*p* < 0.001), despite partial improvement post-treatment (*p* < 0.001).

Overall, only the clove, F+nHAp, and nHAp groups achieved microhardness values after remineralization that were statistically indistinguishable from their baseline values, suggesting superior remineralization capacity.

### SEM analysis findings

**Fig. 4:**
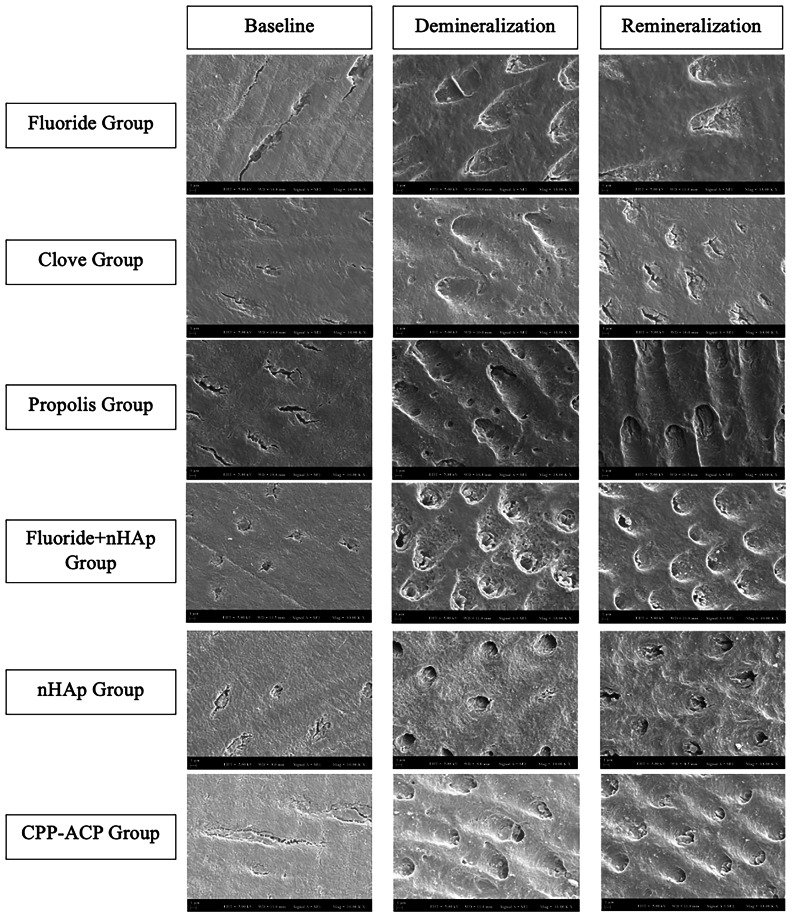
SEM micrographs of dentin surfaces at ×5000 magnification.

Scanning electron microscopy (SEM) micrographs of dentin surfaces captured before erosion, after the demineralization cycle, and following remineralization are presented in Fig. 4 at ×5000 magnification.

Baseline SEM images (Fig. 4) showed the presence of a uniform smear layer covering the dentin surface in all experimental groups. Following demineralization (Fig. 4, demineralization), the smear layer was completely removed, exposing open dentinal tubules and indicating pronounced surface erosion. After the remineralization (Fig. 4, remineralization), partial occlusion of the dentinal tubule orifices was observed, suggesting a degree of surface repair associated with the applied toothpaste treatments.

### AFM analysis findings

**Fig. 5:**
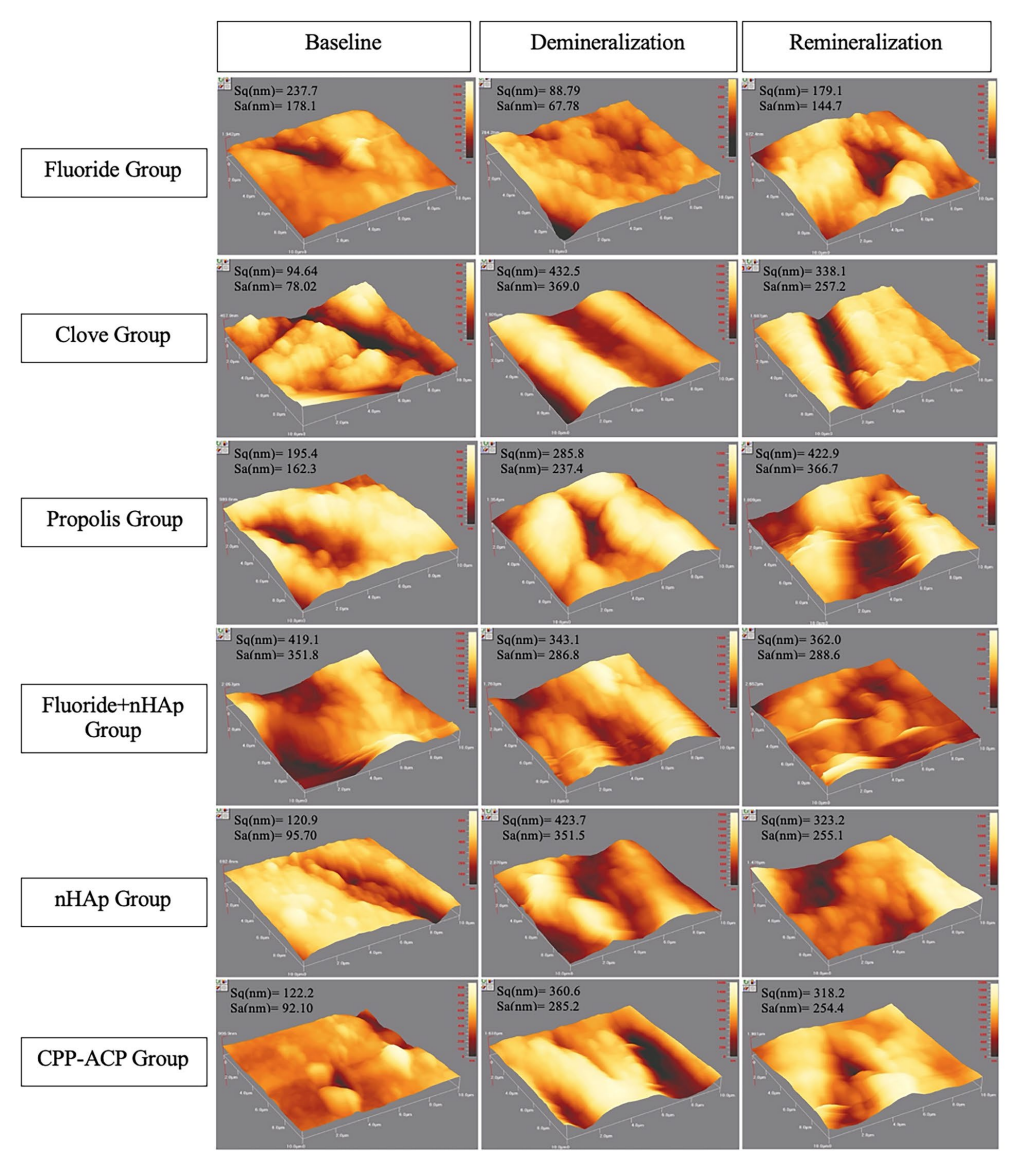
3D images obtained with atomic force microscopy.

Atomic force microscopy (AFM) images revealed that the baseline dentin surfaces across all groups displayed smooth and uniform topographies. Following demineralization, all groups exhibited comparable surface morphologies with increased roughness relative to baseline. After remineralization, the clove, CPP–ACP, and fluoride groups showed surface features closely resembling their baseline conditions, with visibly reduced roughness. In contrast, the propolis, F+nHAp, and nHAp groups displayed higher surface roughness, suggesting less effective surface recovery in these formulations (Fig. 5).

## Discussion

This study investigated the in vitro protective effects of various herbal toothpaste formulations on dentin erosion, focusing on surface roughness and microhardness parameters. The findings revealed that the surface roughness of erosive dentin increased in all groups following both demineralization and remineralization procedures. Additionally, all groups exhibited a statistically significant increase in microhardness values after the remineralization phase compared to the post-demineralization measurements (*p* < 0.05). Based on these findings, the null hypothesis was rejected.

Dental erosion, characterized by the progressive and irreversible loss of hard dental tissues due to acid exposure without bacterial involvement, has been simulated using diverse acid protocols^23^. Citric acid (pH 1.0–3.2) is typically used to mimic dietary acids, whereas hydrochloric acid (pH 1.5), as in the present study, simulates intrinsic factors such as gastric reflux^24,25^. Based on previous literature, a three-day erosion cycle was employed, aligning with commonly reported durations of two to six days^16,26^.

Among various analytical techniques such as confocal laser scanning microscopy, transverse microradiography, and scanning electron microscopy; surface profilometry was selected due to its reproducibility, ease of use, and high sensitivity in detecting micrometer-level surface loss^27^. The vickers microhardness test, known for its non-destructive nature and reliability, was also utilized to assess remineralization effects^28^.

In this study, surface roughness and microhardness measurements, along with AFM and SEM analyses, were employed to assess the effects of toothpaste abrasivity on eroded surfaces. Each specimen exposed to the erosive cycle was brushed with an electric toothbrush, applying a constant force of 2 N for a total duration of nine minutes. This brushing protocol was selected to closely simulate clinical conditions. Several studies have indicated that, although toothbrushing duration varies across individuals, the actual average brushing time is approximately 120 s. This equates to roughly 2 s of brushing per tooth surface per day^22^. Therefore, to simulate approximately four months of toothbrushing, the specimens were brushed for a total of nine minutes as part of the brushing simulation protocol.

In one study, various treatment modalities (application of fluoride varnish, brushing with desensitizing toothpaste, and diode laser treatment) were evaluated on bovine root dentin specimens. After treatment, surface roughness was analyzed, and the specimens were subsequently incubated with *Streptococcus mutans* to assess bacterial adhesion. The study’s findings revealed that brushing with desensitizing toothpaste resulted in the highest level of bacterial accumulation on root dentin surfaces. Moreover, bacterial adhesion was found to significantly increase on dentin surfaces exhibiting roughness values exceeding 0.2–0.3 μm following the erosive cycle^29^. Previous studies have reported that toothpaste use may result in an eight-fold increase in surface roughness^30^. In the present study, surface roughness increased approximately threefold relative to baseline levels after toothbrushing and exposure to an erosive challenge.

The roughness values of sound and eroded dentin obtained in this study are in agreement with the results of previous studies in the literature^31,32^. Toothpastes commonly include abrasive particles, such as silica derivatives, which function as polishing agents^33^. These components are added to toothpaste formulations to eliminate bacterial biofilms and reduce residual deposits on tooth surfaces. However, by increasing the abrasiveness of the toothpaste mixture, which is measured by relative dentin abrasivity (RDA), they may contribute to dentin wear. The surface roughness of the tested toothpastes on eroded dentin was found to be higher than that on sound dentin^33,34^.

The abrasive effect of toothpastes in vitro may be influenced by the number of brushing cycles used in experimental protocols. However, previous studies have shown that dentin loss does not increase proportionally with the number of brushing strokes. Moore and Addy^35^ reported that dentine loss was not proportional to brushing cycles, with less additional tissue loss observed after the second 10,000 strokes compared with the first cycle. Similarly, Johannsen et al.^33^ demonstrated that abrasion was not linearly related to brushing time or stroke count. Therefore, this methodological variable should be considered when comparing results among different in vitro studies.

Tooth surfaces exposed to acidic challenges become increasingly vulnerable to mineral loss due to the influence of abrasive factors, such as brushing and toothpaste^36^.

In the present study, surface roughness increased significantly in all groups after demineralization. Following the remineralization phase, the clove group exhibited surface roughness values that were most similar to the baseline measurements. In contrast, roughness values remained elevated compared to baseline in all other groups. Among all groups, the propolis group exhibited the highest post-remineralization surface roughness, whereas the clove group showed the lowest values (Fig. 5). Statistically, surface roughness after remineralization was significantly higher than the baseline values in all groups except the clove group (*p* < 0.05). Contrary to expectations based on formulation content, the clove group exhibited the lowest surface roughness values following the remineralization phase, despite containing hydrated silica, a commonly used abrasive agent. In contrast, the CPP–ACP group, which lacks hydrated silica, showed comparatively higher surface roughness. Notably, a statistically significant difference was observed between the clove and propolis groups in the post-remineralization phase (*p* < 0.05), with the propolis group exhibiting the highest roughness value among all groups.

This finding deviates from conventional assumptions in the literature, which typically associate increased roughness with silica-containing formulations due to their polishing effects and abrasive potential^1^. The performance of the tested toothpastes may also be influenced by interactions between their formulation components. Commercial dentifrices typically contain multiple constituents, including abrasive agents, humectants, surfactants, and stabilizers, in addition to their primary active ingredients, and these components may collectively influence their effects on dentin surfaces^33^. Abrasive particles such as hydrated silica contribute to cleaning efficiency and stain removal; however, they may also increase dentin wear depending on their concentration and physicochemical properties^1,33^. Previous studies have demonstrated that toothpaste abrasivity is not determined solely by the presence of abrasive agents but is also affected by particle characteristics such as size, shape, and hardness^33^. Furthermore, bioactive ingredients such as calcium–phosphate–based compounds or plant-derived components may interact with dentin surfaces and potentially influence remineralization processes^9,13^. Therefore, the observed outcomes likely reflect the combined influence of multiple formulation components rather than the isolated effect of a single active ingredient^1^.

The reduced surface roughness observed in the clove group may be attributed to the presence of eugenol and other antioxidant-rich constituents, such as tocopherol and thymol, which have been reported to exert anti-inflammatory and reparative effects on both soft and hard oral tissues^37,38^. These components may have supported surface recovery, potentially counteracting the abrasive effect of hydrated silica. Conversely, the increased surface roughness in the CPP–ACP group despite the absence of abrasive agents may be explained by the limited mechanical cohesion of the CPP–ACP matrix under acidic stress or inadequate surface retention during brushing^9^. These formulation-dependent interactions warrant further investigation in future studies.

Microhardness analysis revealed a statistically significant decrease in all toothpaste groups following demineralization compared to baseline values (*p* < 0.05), indicating mineral loss due to acidic erosion. These findings align with previous studies, including those by Carey and Brown, which reported similar demineralization effects under acidic conditions^24^. After remineralization, the highest microhardness values were observed in the F+nHAp and nHAp groups (60.87 ± 3.18 and 60.52 ± 4.44, respectively), both significantly higher than those in the other groups.

Following remineralization, significant increases in microhardness values were observed in the clove, F+nHAp, and nHAp groups (*p* < 0.05), indicating their effectiveness in enhancing dentin hardness after erosive and abrasive challenges.

The increased dentin hardness observed in the clove group may be associated with the high eugenol content (81.1%) of clove extract, a compound known for its biological activity^39^. A study by Mendi et al.^38^ reported that extracts of clove, cinnamon, and sage promote both the proliferation and osteogenic differentiation of dental pulp stem cells. Specifically, clove extract was found to enhance osteocalcin expression and stimulate calcium granule formation, both of which are essential for hard tissue regeneration^14,40^. Additionally, tocopherol, another bioactive compound present in the clove formulation, has been reported to improve microhardness on demineralized enamel surfaces due to its antioxidant and reparative properties^41^.

Following the remineralization phase, the clove, F+nHAp, and nHAp groups exhibited microhardness values statistically similar to their baseline levels, indicating their potential in the treatment of eroded or abraded dentin. The favorable outcome in these groups may be attributed to the nano-hydroxyapatite content in F+nHAp and nHAp, as well as to bioactive agents such as eugenol and tocopherol in the clove formulation.

The near-baseline surface hardness values observed in the clove group suggest a potential for clove-containing toothpaste to enhance mineral deposition following erosion, thereby supporting the restoration of dentin integrity (Fig. 4).

Although hydroxyapatite nanoparticles (nano-HA) are known to penetrate demineralized dentin and act as scaffolds for remineralization, data on their interaction with nano-HA-containing toothpastes remain limited^42,43^. Leal et al.^44^ evaluated an experimental dentifrice containing both high-concentration fluoride (5,000 µg/g) and nano-HA, reporting a significant reduction in root dentin demineralization. Importantly, nano-HA alone was also effective in reducing demineralization, suggesting that its protective effect is not solely dependent on fluoride. Despite these findings, few studies have explored the synergistic potential of combining fluoride and nano-HA in commercial formulations. Nano-HA particles have been demonstrated to act as reservoirs for calcium and phosphate ions, effectively occluding dentinal tubules and thereby supporting remineralization through both chemical and physical mechanisms^45,46^.

Interestingly, the F+nHAp and nHAp groups showed similar performance, suggesting that the addition of fluoride may not confer a significant additional benefit in this formulation. However, this observation warrants further investigation to assess potential synergistic effects. Consistently, SEM images obtained after remineralization in the F+nHAp and nHAp groups revealed partial occlusion of dentinal tubules (Fig. 4). Furthermore, the lack of a statistically significant difference between post-remineralization and baseline microhardness values further supports this finding (Table 3).

The inability of the fluoride, propolis, and CPP–ACP toothpaste groups to restore microhardness to baseline levels suggests a limited remineralization capacity for these formulations. Fluoride-containing products are known to promote remineralization by forming a mineral-rich layer on the tooth surface, thereby reducing demineralization and aiding in the management of dental erosion^18^. However, the protective effect of fluoride alone is generally restricted to enamel and its subsurface layers, with reduced efficacy on exposed dentin surfaces^47^. This may explain the suboptimal outcomes observed in these groups following the remineralization phase.

In this study, the propolis group did not show a significant increase in microhardness values after remineralization compared to baseline. However, Naghsh et al.^15^ demonstrated that propolis-containing toothpaste effectively occludes dentinal tubules, significantly reducing the proportion of open tubules. This suggests that while propolis may not contribute meaningfully to structural remineralization, it may still play a role in reducing dentin hypersensitivity through tubule occlusion. These findings suggest that the functional benefits of propolis may be formulation-dependent, highlighting the need for further research to clarify its role in dentin protection.

In this study, the fluoride group exhibited a significant increase in surface roughness compared to baseline, both after demineralization and following the remineralization phase (*p* < 0.05). Additionally, although the abrasive effects of toothpaste are believed to be influenced by the number of brushing cycles, no consistent linear correlation has been established between cycle count and tissue loss^33^. This variability should be taken into account when comparing findings across different studies.

Nevertheless, the primary limitation of this study is its in vitro design, which restricts the extrapolation of results to clinical conditions. Variability in intraoral pH dynamics, salivary composition, and brushing behaviors among individuals may influence the real-world performance of toothpaste formulations. Furthermore, the standardized brushing protocol cannot fully replicate long-term patient-specific usage. In addition, the absence of a negative control group (e.g., brushing with distilled water or an inert base formulation without active agents) limits the ability to completely isolate the mechanical contribution of brushing from the chemical effects of the tested formulations. Therefore, the observed outcomes reflect the combined influence of mechanical brushing and formulation-specific components.

Another limitation of the present study is that the Relative Dentin Abrasivity (RDA) values were not available for all tested toothpastes. Since abrasivity may influence dentin surface alterations during erosive–abrasive challenges, the inability to report RDA values for some formulations limits the direct comparison of their abrasivity profiles.

Future clinical trials involving diverse populations and extended evaluation periods are necessary to validate the efficacy of herbal dentifrices under physiological conditions.

## Conclusion

This study demonstrated that specific herbal and nano-hydroxyapatite-containing toothpaste formulations provide measurable protective effects against dentinal erosion, as shown by improvements in surface roughness and microhardness. Among the tested formulations, the clove-containing and nano-hydroxyapatite-based toothpastes were the most effective in preserving dentin integrity. These findings support the potential use of biocompatible herbal toothpastes as adjuncts in the prevention of erosive tooth wear. Further in vivo investigations are warranted to confirm their long-term effectiveness and safety in clinical practice.

## Data Availability

The datasets generated and/or analyzed during the current study are available in the Zenodo repository, https://doi.org/10.5281/zenodo.17195756.

## References

[1] Shellis, R. P. & Addy, M. The interactions between attrition, abrasion and erosion in tooth wear. *Monogr. Oral Sci.* **25**, 32–45. 10.1159/000359936 (2014). 24993256 10.1159/000359936

[2] Lussi, A. & Jaeggi, T. Erosion–diagnosis and risk factors. *Clin. Oral Investig*. **12** (Suppl 1). 10.1007/s00784-007-0179-z (2008). S5-13. 10.1007/s00784-007-0179-z PMC2238777 18228059

[3] Bartlett, D. Intrinsic causes of erosion. *Monogr. Oral Sci.* **20**, 119–139. 10.1159/000093359 (2006). 16687891 10.1159/000093359

[4] Lussi, A. & Carvalho, T. S. Erosive tooth wear: a multifactorial condition of growing concern and increasing knowledge. *Monogr. Oral Sci.* **25**, 1–15. 10.1159/000360380 (2014). 24993253 10.1159/000360380

[5] Barbour, M. E. et al. The relationship between enamel softening and erosion caused by soft drinks at a range of temperatures. *J. Dent.* **34** (3), 207–213. 10.1016/j.jdent.2005.06.002 (2006). 16112333 10.1016/j.jdent.2005.06.002

[6] Campus, G., Niu, J. Y., Sezer, B. & Yu, O. Y. Prevention and management of dental erosion and decay. *BMC Oral Health*. **24** (1), 468. 10.1186/s12903-024-04257-y (2024). 38632545 10.1186/s12903-024-04257-y PMC11025157

[7] Kanaan, M., Brabant, A., Eckert, G. J., Hara, A. T. & Carvalho, J. C. Non-Biological and Biological Risk Indicators for Tooth Wear Outcomes in Adults. *Caries Res.* **56** (4), 407–418. 10.1159/000527091 (2022). 36116437 10.1159/000527091

[8] Cochrane, N. J. & Reynolds, E. C. Calcium phosphopeptides -- mechanisms of action and evidence for clinical efficacy. *Adv. Dent. Res.* **24** (2), 41–47. 10.1177/0022034512454294 (2012). 22899678 10.1177/0022034512454294

[9] Reynolds, E. C. Calcium phosphate-based remineralization systems: scientific evidence? Aust. *Dent. J.* **53** (3), 268–273. 10.1111/j.1834-7819.2008.00061.x (2008). 10.1111/j.1834-7819.2008.00061.x 18782374

[10] Pepla, E., Besharat, L. K., Palaia, G., Tenore, G. & Migliau, G. Nano-hydroxyapatite and its applications in preventive, restorative and regenerative dentistry: a review of literature. *Ann. Stomatol. (Roma)*. **5** (3), 108–114 (2014). 25506416 PMC4252862

[11] Chen, L. et al. Hydroxyapatite in Oral Care Products-A Review. *Mater. (Basel)*. **14** (17). 10.3390/ma14174865 (2021). 10.3390/ma14174865 PMC8432723 34500955

[12] Anwar, M. A. et al. Herbal remedies for oral and dental health: a comprehensive review of their multifaceted mechanisms including antimicrobial, anti-inflammatory, and antioxidant pathways. *Inflammopharmacology* **33** (3), 1085–1160. 10.1007/s10787-024-01631-8 (2025). 39907951 10.1007/s10787-024-01631-8 PMC11914039

[13] Przybylek, I. & Karpinski, T. M. Antibacterial Properties of Propolis. *Molecules* **24** (11). 10.3390/molecules24112047 (2019). 10.3390/molecules24112047 PMC6600457 31146392

[14] Sarialioglu Gungor, A. & Donmez, N. Dentin erosion preventive effects of various plant extracts: An in vitro atomic force microscopy, scanning electron microscopy, and nanoindentation study. *Microsc Res. Tech.* **84** (5), 1042–1052. 10.1002/jemt.23665 (2021). 33264465 10.1002/jemt.23665

[15] Naghsh, N., Mazrooei, F., Hosseini, A., Kiani, S. & Sahebkar, A. Effects of Propolis-Based Herbal Toothpaste on Dentine Hypersensitivity. *Int. Dent. J.* **74** (3), 559–565. 10.1016/j.identj.2023.11.014 (2024). 38184459 10.1016/j.identj.2023.11.014 PMC11123535

[16] Takeshita, E. M., Danelon, M., Castro, L. P., Cunha, R. F. & Delbem, A. C. Remineralizing Potential of a Low Fluoride Toothpaste with Sodium Trimetaphosphate: An in situ Study. *Caries Res.* **50** (6), 571–578. 10.1159/000449358 (2016). 27811470 10.1159/000449358

[17] Yamaguchi, K., Miyazaki, M., Takamizawa, T., Inage, H. & Moore, B. K. Effect of CPP-ACP paste on mechanical properties of bovine enamel as determined by an ultrasonic device. *J. Dent.* **34** (3), 230–236. 10.1016/j.jdent.2005.06.005 (2006). 16112336 10.1016/j.jdent.2005.06.005

[18] Ne, Y. G. S. et al. Treatment for dental erosion: a systematic review of in vitro studies. *PeerJ* **10**, e13864. 10.7717/peerj.13864 (2022). 36389398 10.7717/peerj.13864 PMC9651041

[19] Dionysopoulos, D. et al. Effect of Whitening Toothpastes with Different Active Agents on the Abrasive Wear of Dentin Following Tooth Brushing Simulation. *J. Funct. Biomater.* **14** (5). 10.3390/jfb14050268 (2023). 10.3390/jfb14050268 PMC10219497 37233378

[20] Pereira, C. K. K. et al. In vitro effect of toothpaste with low fluoride combined with sodium trimetaphosphate on dentine erosion. *Eur. Arch. Paediatr. Dent.* **22** (5), 843–849. 10.1007/s40368-021-00636-z (2021). 34056698 10.1007/s40368-021-00636-z

[21] Tezvergil-Mutluay, A. et al. Effects of Composites Containing Bioactive Glasses on Demineralized Dentin. *J. Dent. Res.* **96** (9), 999–1005. 10.1177/0022034517709464 (2017). 28535357 10.1177/0022034517709464

[22] Jasse, F. F. et al. Influence of filler charge on gloss of composite materials before and after in vitro toothbrushing. *J. Dent.* **41** (Suppl 5), e41–e44. 10.1016/j.jdent.2013.04.011 (2013). 23649047 10.1016/j.jdent.2013.04.011

[23] Barac, R. et al. In vitro effect of beer, red and white wine on the morphology and surface roughness of human enamel. *Adv. Clin. Exp. Med.* **32** (11), 1241–1248. 10.17219/acem/161856 (2023). 37077143 10.17219/acem/161856

[24] Carey, C. M. & Brown, W. Dentin Erosion: Method Validation and Efficacy of Fluoride Protection. *Dent. J. (Basel)*. **5** (4). 10.3390/dj5040027 (2017). 10.3390/dj5040027 PMC5806968 29563433

[25] Batista, G. R., Rocha Gomes Torres, C., Sener, B., Attin, T. & Wiegand, A. Artificial Saliva Formulations versus Human Saliva Pretreatment in Dental Erosion Experiments. *Caries Res.* **50** (1), 78–86. 10.1159/000443188 (2016). 26870948 10.1159/000443188

[26] Scaramucci, T., Borges, A. B., Lippert, F., Zero, D. T. & Hara, A. T. In vitro effect of calcium-containing prescription-strength fluoride toothpastes on bovine enamel erosion under hyposalivation-simulating conditions. *Am. J. Dent.* **28** (1), 18–22 (2015). 25864237

[27] Schwendicke, F., Felstehausen, G., Carey, C. & Dorfer, C. Comparison of four methods to assess erosive substance loss of dentin. *PLoS One*. **9** (9), e108064. 10.1371/journal.pone.0108064 (2014). 25229410 10.1371/journal.pone.0108064 PMC4168231

[28] Sakar-Deliormanli, A. & Guden, M. Microhardness and fracture toughness of dental materials by indentation method. *J. Biomed. Mater. Res. B Appl. Biomater.* **76** (2), 257–264. 10.1002/jbm.b.30371 (2006). 16211564 10.1002/jbm.b.30371

[29] Cury, M. S. et al. Surface roughness and bacterial adhesion on root dentin treated with diode laser and conventional desensitizing agents. *Lasers Med. Sci.* **33** (2), 257–262. 10.1007/s10103-017-2356-x (2018). 29032514 10.1007/s10103-017-2356-x

[30] Ramos, F. S. et al. Effect of different toothpastes on permeability and roughness of eroded dentin. *Acta Odontol. Latinoam.* **35** (3), 229–237. 10.54589/aol.35/3/229 (2022). 36748742 10.54589/aol.35/3/229 PMC10283392

[31] Zanatta, R. F. et al. Protection of calcium silicate/sodium phosphate/fluoride toothpaste with serum on enamel and dentin erosive wear. *J. Appl. Oral Sci.* **29**, e20210081. 10.1590/1678-7757-2021-0081 (2021). 34614120 10.1590/1678-7757-2021-0081 PMC8523098

[32] Memarpour, M., Jafari, S., Rafiee, A., Alizadeh, M. & Vossoughi, M. Protective effect of various toothpastes and mouthwashes against erosive and abrasive challenge on eroded dentin: an in vitro study. *Sci. Rep.* **14** (1), 9387. 10.1038/s41598-024-59631-1 (2024). 38653765 10.1038/s41598-024-59631-1 PMC11039751

[33] Johannsen, G., Tellefsen, G., Johannsen, A. & Liljeborg, A. The importance of measuring toothpaste abrasivity in both a quantitative and qualitative way. *Acta Odontol. Scand.* **71** (3–4), 508–517. 10.3109/00016357.2012.696693 (2013). 22746180 10.3109/00016357.2012.696693 PMC3665314

[34] Vertuan, M., de Souza, B. M., Machado, P. F., Mosquim, V. & Magalhaes, A. C. The effect of commercial whitening toothpastes on erosive dentin wear in vitro. *Arch. Oral Biol.* **109**, 104580. 10.1016/j.archoralbio.2019.104580 (2020). 31593890 10.1016/j.archoralbio.2019.104580

[35] Moore, C. & Addy, M. Wear of dentine in vitro by toothpaste abrasives and detergents alone and combined. *J. Clin. Periodontol*. **32** (12), 1242–1246. 10.1111/j.1600-051X.2005.00857.x (2005). 16269001 10.1111/j.1600-051X.2005.00857.x

[36] Carvalho, T. S. & Lussi, A. Combined effect of a fluoride-, stannous- and chitosan-containing toothpaste and stannous-containing rinse on the prevention of initial enamel erosion-abrasion. *J. Dent.* **42** (4), 450–459. 10.1016/j.jdent.2014.01.004 (2014). 24440712 10.1016/j.jdent.2014.01.004

[37] Vargas Fda, S., Soares, D. G., Basso, F. G., Hebling, J. & Costa, C. A. Dose-response and time-course of alpha-tocopherol mediating the cytoprotection of dental pulp cells against hydrogen peroxide. *Braz Dent. J.* **25** (5), 367–371. 10.1590/0103-6440201302434 (2014). 25517769 10.1590/0103-6440201302434

[38] Mendi, A. et al. Effects of Syzygium aromaticum, Cinnamomum zeylanicum, and Salvia triloba extracts on proliferation and differentiation of dental pulp stem cells. *J. Appl. Oral Sci.* **25** (5), 515–522. 10.1590/1678-7757-2016-0522 (2017). 29069149 10.1590/1678-7757-2016-0522 PMC5804388

[39] Banerjee, S., Panda, C. K. & Das, S. Clove (Syzygium aromaticum L.), a potential chemopreventive agent for lung cancer. *Carcinogenesis* **27** (8), 1645–1654. 10.1093/carcin/bgi372 (2006). 16501250 10.1093/carcin/bgi372

[40] Bakhori, S. K. M., Mahmud, S., Mohamad, D., Masudi, S. M. & Seeni, A. Surface morphological and mechanical properties of zinc oxide eugenol using different types of ZnO nanopowder. *Mater. Sci. Eng. C Mater. Biol. Appl.* **100**, 645–654. 10.1016/j.msec.2019.03.034 (2019). 30948101 10.1016/j.msec.2019.03.034

[41] Srivastava, M. & Yeluri, R. The effect of 10% alpha-tocopherol solution and 5% grape seed extract on the microhardness and shear bond strength to bleached dentin. *Dent. Res. J. (Isfahan)*. **18**, 54 (2021). 34497689 PMC8404561

[42] Earl, J. S., Wood, D. J. & Milne, S. J. Nanoparticles for dentine tubule infiltration: an in vitro study. *J. Nanosci. Nanotechnol*. **9** (11), 6668–6674. 10.1166/jnn.2009.1330 (2009). 19908582 10.1166/jnn.2009.1330

[43] Besinis, A., van Noort, R. & Martin, N. Infiltration of demineralized dentin with silica and hydroxyapatite nanoparticles. *Dent. Mater.* **28** (9), 1012–1023. 10.1016/j.dental.2012.05.007 (2012). 22721698 10.1016/j.dental.2012.05.007

[44] Leal, A. M. C. et al. Development of an Experimental Dentifrice with Hydroxyapatite Nanoparticles and High Fluoride Concentration to Manage Root Dentin Demineralization. *Int. J. Nanomed.* **15**, 7469–7479. 10.2147/IJN.S264754 (2020). 10.2147/IJN.S264754 PMC7547140 33116482

[45] Bologa, E. S. S., Iovan, G., Ghiorghe, C. A. & Nica, I. Effects of Dentifrices Containing Nanohydroxyapatite on Dentinal Tubule Occlusion—A Scanning Electron Microscopy and EDX Study. *Appl. Sci.* **10** (18), 6513. 10.3390/app10186513 (2020).

[46] Amaechi, B. T., Mathews, S. M., Ramalingam, K. & Mensinkai, P. K. Evaluation of nanohydroxyapatite-containing toothpaste for occluding dentin tubules. *Am. J. Dent.* **28** (1), 33–39 (2015). 25864240

[47] Lussi, A. & Carvalho, T. S. The future of fluorides and other protective agents in erosion prevention. *Caries Res.* **49** (Suppl 1), 18–29. 10.1159/000380886 (2015). 25871415 10.1159/000380886

